# Supplementary material for: Electronic Transport Properties of Silicane Determined from First Principles
Source: Materials (Basel). 2019 Sep 11;12(18):2935. doi: 10.3390/ma12182935 (PMC6766188; doi:10.3390/ma12182935)
Supplement: Supplementary file 1 [file materials-12-02935-s001.pdf]

materials

MDPI

Supplementary

Electronic Transport Properties of Silicane Determined from First Principles

Mohammad Mahdi Khatami [1,2], Gautam Gaddemane [1], Maarten L. Van de Put [1], Massimo V. Fischetti [1], Mohammad Kazem Moravvej-Farshi [2], Mahdi Pourfath [3] and William G. Vandenberghe [1,*]

1 Department of Material science and Engineering, The University of Texas at Dallas, Richardson, Texas 75080, USA
2 Faculty of Electrical and Computer Engineering, Tarbiat Modares University, Tehran 1411713116, Iran
3 School of Electrical and Computer Engineering, University of Tehran, Tehran 14395-515, Iran
* Correspondence: William.Vandenberghe@utdallas.edu; Tel.: 1-972-883-5793

Received: 8 August 2019; Accepted: 6 September 2019; Published: September 2019

We provide the input files used for the Quantum espresso self-consistent runs (scf.in), the phonon calculations (ph.in), the input for the transformation of the force constants to real space (q2r.in), the input file for the non-selfconsistent run (nscf.in), and the input file for the electron-phonon Wannier (EPW) code.

Self-consistent calculations file (scf.in):

```
&CONTROL
  calculation = 'scf' ,
  prefix = 'Silicane' ,
  wf_collect = .true.
  outdir = './' ,
  pseudo_dir = './' ,
  etot_conv_thr = 1.0d-6 ,
/
&SYSTEM
  ibrav = 0,
  a = 3.88717,
  nat = 4,
  ntyp = 2,
  ecutwfc = 50.0,
 /
&ELECTRONS
  conv_thr = 1.0d-12 ,
  mixing_beta = 0.7 ,
 /
 &IONS
 /
 &CELL
 /
CELL_PARAMETERS alat
     0.500000000     0.866025404     0.000000000
    −0.500000000     0.866025404     0.000000000
     0.000000000     0.000000000     5.144000000
ATOMIC_SPECIES
   Si    28.08550   Si.upf
```

```
    H         1.008      H.upf
ATOMIC_POSITIONS crystal
    H         0.000000000      0.000000000      0.000000000
    Si        0.000000000      0.000000000      0.075118000
    Si        0.333333333      0.333333333      0.111100000
    H         0.333333333      0.333333333      0.186218000
K_POINTS automatic
24 24 1 0 0 0
```

© 2019 by the authors. Submitted for possible open access publication under the terms and conditions of the Creative Commons Attribution (CC BY) license (http://creativecommons.org/licenses/by/4.0/).
